# Supplementary material for: Solute Carrier Family 29A1 Mediates In Vitro Resistance to Azacitidine in Acute Myeloid Leukemia Cell Lines
Source: Int J Mol Sci. 2023 Feb 10;24(4):3553. doi: 10.3390/ijms24043553 (PMC9965596; doi:10.3390/ijms24043553)
Supplement: Supplementary file 1 [file ijms-24-03553-s001.zip › ijms-2202586-supplementary.pdf]

Supplementary Information

This section includes:

1. **Tables S1 - S3**

2. **Supplementary Figures S1 – S6**

Supplementary Tables

Table S1. AZA resistant cell lines nomenclature

Parental Cell line	Cell Line Name	Initial AZA Concentration (µM)	Maximal AZA Concentration (µM)	Minimum Days in indicated AZA Concentration	Cumulative Time (Months) to Resistance Development
**MOLM-13**	**M-Naïve**	0	0	0	N/A - sensitive
	**M-R0.4**	0.1	0.4	15	1.6
	**M-R1**		1	15	2
	**M-R5**		5	14	3.25
	**M-R10**		10	14	3.6
**SKM-1**	**S-Naïve**	0	0	0	N/A - sensitive
	**S-R0.4**	0.1	0.4	14	2
	**S-R1**		1	21	2.75
	**S-R5**		5	14	7.4
	**S-R10**		10	14	8.3

Table S2. Cytogenetics of MOLM-13 and SKM-1 Naïve and azacitidine resistant cells

Cell line	Karyotype
**MOLM-13**	
Naïve	52,XY,+6,+8,+8,+8,add(8)(p11.2),ins(11;9)(q23;p22p23),+13,t(17;20)(p11.2;q11.2),+19[30]
R0.4	52,XY,+6,+8,+8,+8,add(8)(p11.2),ins(11;9)(q23;p22p23),+13,t(17;20)(p11.2;q11.2),+19[23]
R1	52,XY,+6,+8,+8,+8,add(8)(p11.2),ins(11;9)(q23;p22p23),+13,t(17;20)(p11.2;q11.2),+19[25]
R5	52,XY,+6,+8,+8,+8,add(8)(p11.2),ins(11;9)(q23;p22p23),+13,t(17;20)(p11.2;q11.2),+19[20]
**SKM-1**	
Naïve	43,XY,del(2)(p11.2p23),add(7)(34),del(9)(q13q34),-10,-12,-14,add(17)(p13),der(19)t(1;19)(q12;q13.3),add(20)(p13),der(21)t(10;21)q11.2;p11.2)[39]
R0.4	43,XY,del(2)(p11.2p23),add(7)(34),del(9)(q13q34),-10,-12,-14, add(17)(p13),der(19)t(1;19)(q12;q13.3), add(20)(p13),der(21)t(10;21)q11.2;p11.2)[12]/43,idem,del(1)(q21)[2]
R1	43,XY,del(2)(p11.2p23),add(7)(34),del(9)(q13q34),-10,-12,-14, add(17)(p13), der(19)t(1;19)(q12;q13.3), add(20)(p13),der(21)t(10;21)q11.2;p11.2)/43,idem,del(1)(q?21)
R5	43,XY,del(2)(p11.2p23),add(7)(34),del(9)(q13q34),-10,-12,-14,add(17)(p13),der(19)t(1;19)(q12;q13.3), add(20)(p13),der(21)t(10;21)q11.2;p11.2)[1]/43,sl,del(1)(q21)[31]

Table S3. The potential targets of selected inhibitors for ATP-Binding cassette and solute carrier proteins.

Inhibitor	Target		
	Efflux	Influx	Bi-directional
**NBMPR**	No evidence	SLC29A1, SLC29A2 (moderate), SLC29A3 (moderate), SLC29A4 (moderate)	No evidence
**Verapamil**	ABCB1, ABCB4, ABCB11, ABCC1, ABCC2, ABCC3, ABCC4	SLC22A1, SLC22A2, SLC22A4, SLC22A5, SLCO1A2, SLCO1B1	SLC47A1, SLC47A2
**Chloroquine**	ABCB1, ABCB11, ABCC2, ABCC3, ABCC4, ABCG2	SLC22A1, SLC22A2, SLCO1A2	SLC47A1, SLC47A2
**Cimetidine**	ABCB1, ABCB11, ABCC2, ABCC3, ABCC4	SLC22A1, SLC22A2, SLC22A4, SLC22A8	SLC47A1, SLC47A2
**Amantadine**	No evidence	SLC22A1, SLC22A2, SLC22A3	SLC47A1, SLC47A2

**Procainamide**	ABCB11, ABCC2, ABCC3, ABCC4	SLC22A1, SLC22A2, SLC22A3, SLC22A4, SLC22A5	SLC47A1, SLC47A2
**Cyclosporine**	ABCB1, ABCB4, ABCB11, ABCC1, ABCC2, ABCC3, ABCC4, ABCG2	SLCO1B3, SLC10A1, SLCO1B1	No evidence
**Pyrimethamine**	No evidence	SLC22A1, SLC22A2	SLC47A1, SLC47A2
**Corticosterone**	No evidence	SLC22A1, SLC22A2	No evidence

Targets in red are the targets significantly differently expressed in our cohort.

Supplementary Figures

Figure S1.

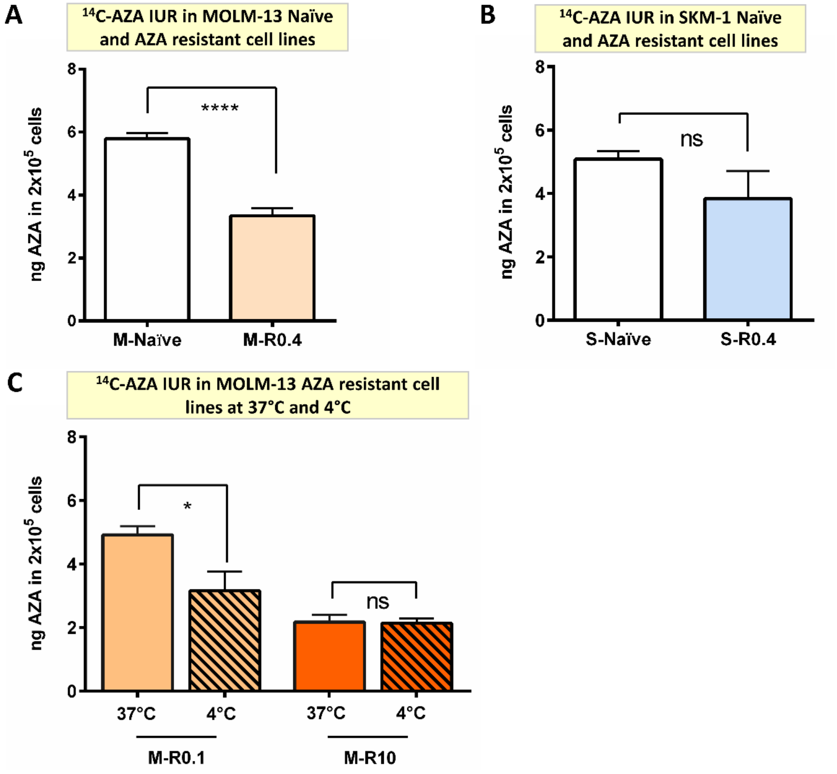

Figure S1. The impact of temperature on ^{14}C-AZA intracellular uptake and retention (IUR) in resistant cells. (A) ^{14}C-AZA IUR was significantly lower in MOLM-13 resistant cells (M-R0.4) compared to parent cells; (B) although ^{14}C-AZA IUR is lower in SKM-1 resistant cells (S-R0.4) compared to parent cells, it was not statistically significant; (C) In MOLM-13 cells with minimal resistance to AZA (M-R0.1), ^{14}C-AZA IUR was significantly lower at 4^{0}C compared to 37^{0}C suggesting temperature dependent active cellular transport. ^{14}C-AZA IUR was significantly lower in highly resistant (M-R10) cells compared to M-R0.1, importantly, effect of temperature was lost in M-R10. Data represent the mean and all error bars indicate SEM of at least 3 independent experiments. AZA, Azacitidine; M, MOLM-13; S, SKM-1. Unpaired Student's t-test (Welch's correction was applied for data groups with unequal SD) was used to detect

statistically significant differences between cohorts. Asterisks display *P*-values *P < 0.05, ****P < 0.0001.

Figure S2.

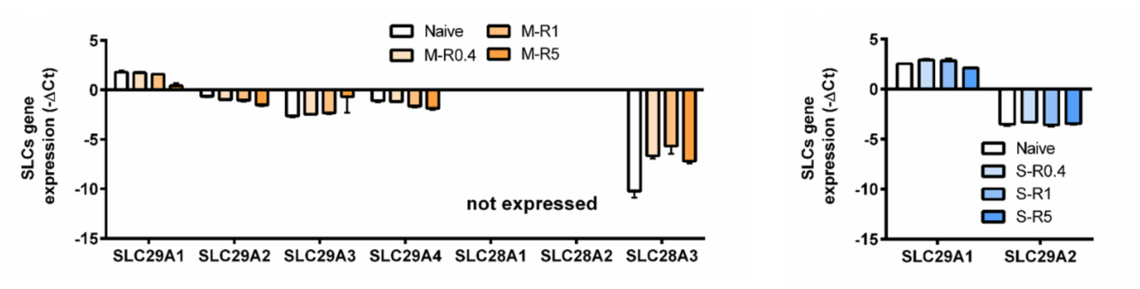

(**A**) (**B**)

Figure S2. The expression of *SLC29* and *SLC28* genes families in MOLM-13 and SKM-1 AZA Naïve and resistant cell lines. (**A**) *SLC29A1* mRNA expression is higher in MOLM-13 Naïve and resistant cells compared to other *SLC29A* and *SLC28A* gene expression; (**B**) similarly *SLC29A1* expression was higher compared to *SLC29A2* in SKM-1 Naïve and resistant cells.

Figure S3.

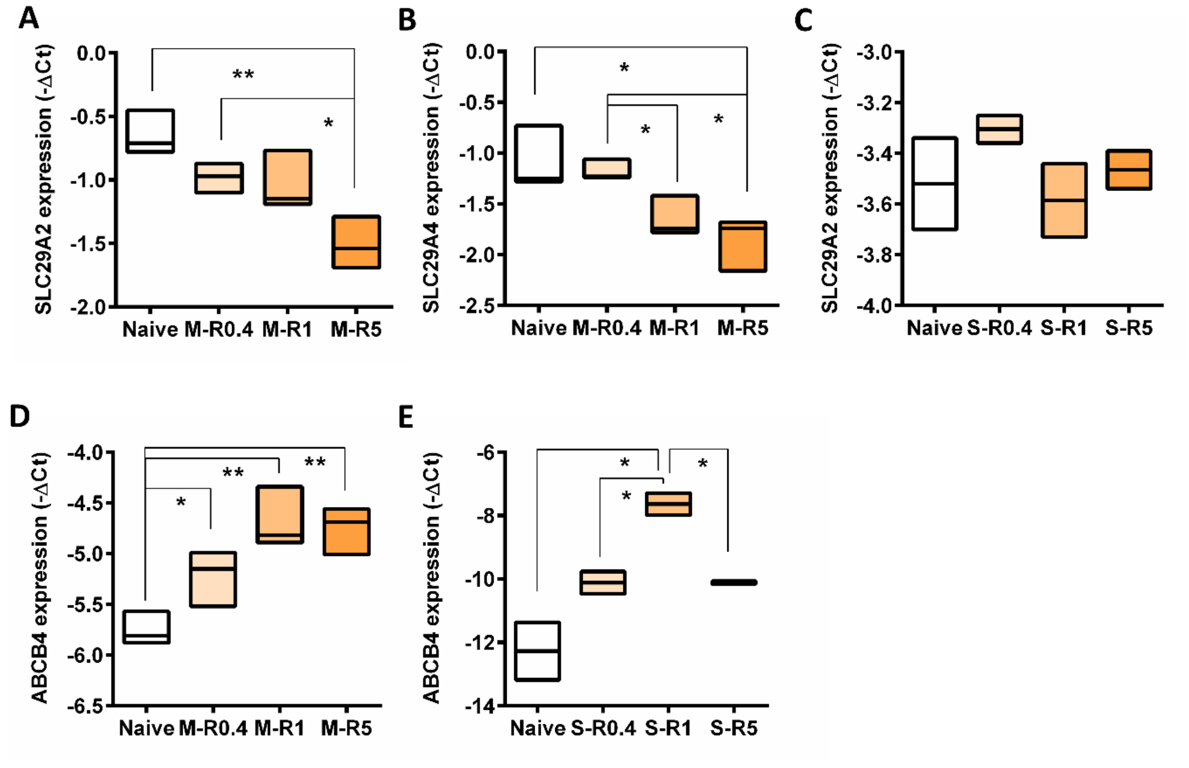

Figure S3. Box plot representing gene expression of selected genes in Naïve and AZA resistant cell lines, (A) *SLC29A2* and (B) *SLC29A4* in MOLM-13, (C) *SLC29A2* in SKM-1, (D-E) *ABCB4* in MOLM-13 and SKM-1 respectively**.** Student's t-test was used to detect statistically significant differences between cohorts. Asterisks display *P*-values * $p < 0.05$, ** $p < 0.01$.

Figure S4.

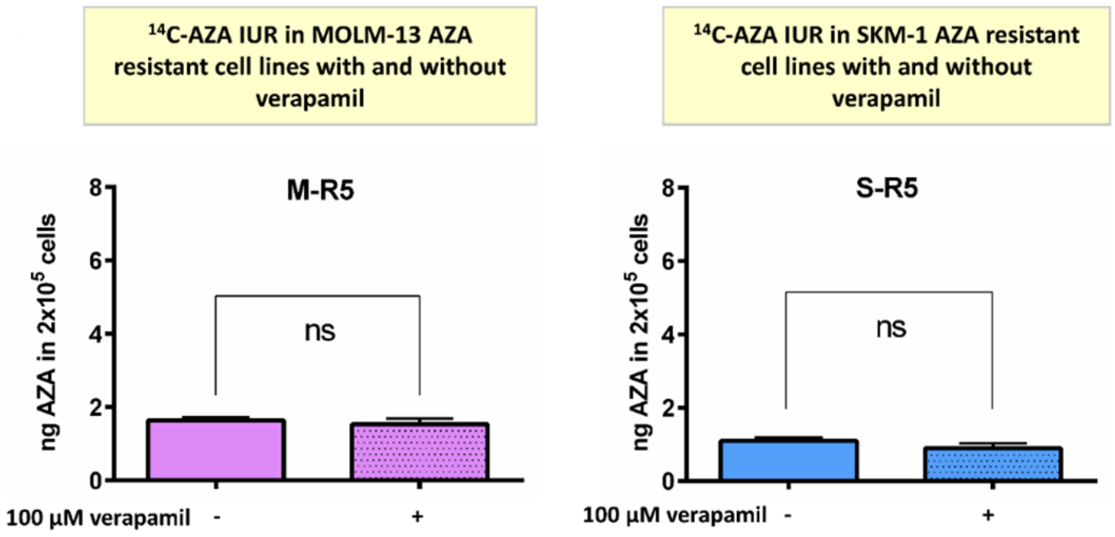

(**A**) (**B**)

Figure S4. ABCB4 inhibitor verapamil did not influence ^{14}C-AZA IUR in MOLM-13 and SKM-1 AZA resistant cell lines. ^{14}C-AZA IUR in (**A**) MOLM-13 (**B**) SKM-1 AZA resistant cell lines with and without verapamil. Data represent the mean and all error bars indicate SEM of at least 3 independent experiments. AZA, Azacitidine; M, MOLM-13; S, SKM-1. Unpaired Student's t-test (Welch's correction was applied for data groups with unequal SD) was used to detect statistically significant differences between cohorts.

Figure S5.

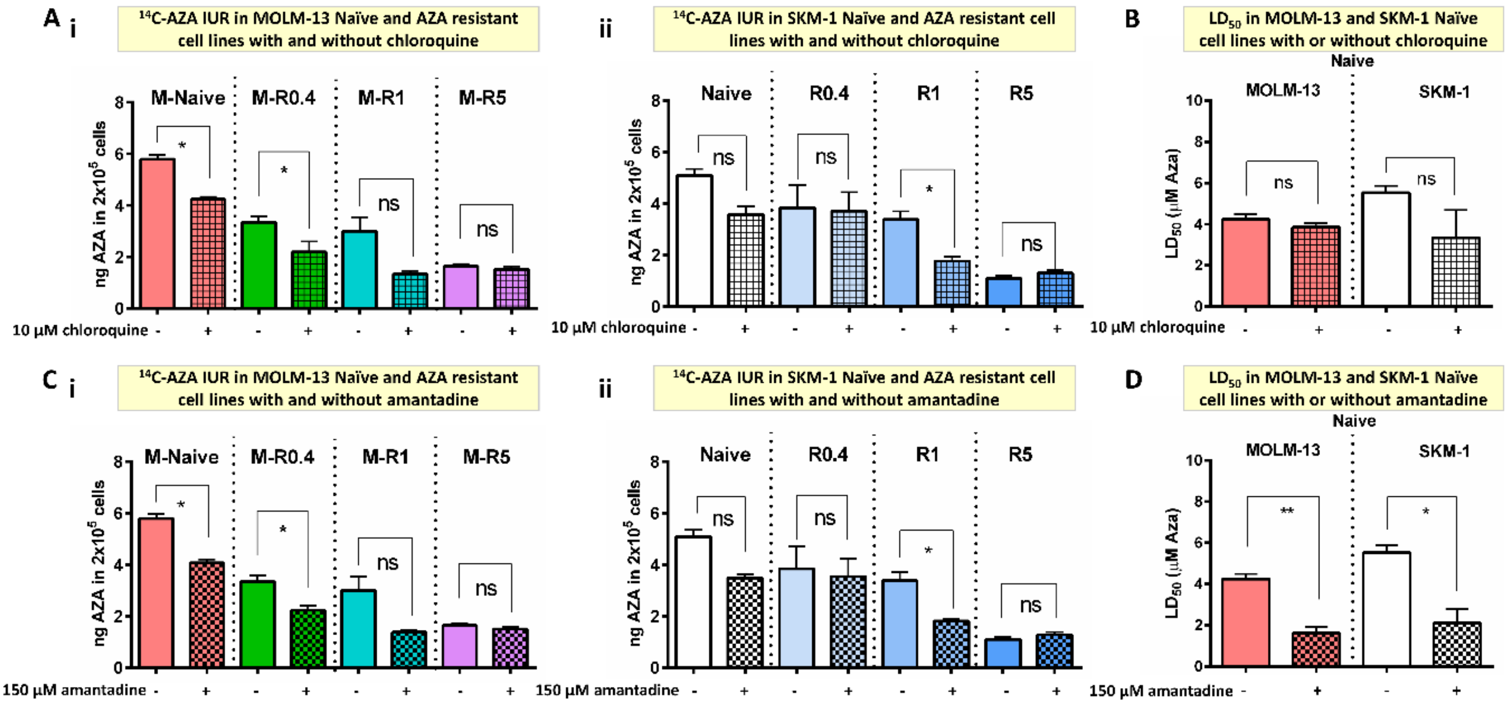

Figure S5. The effects of chloroquine and amantadine on ^{14}C-AZA IUR in Naïve and AZA resistant cell lines across MOLM-13 and SKM-1. (A) ^{14}C-AZA IUR in (i) MOLM-13 (ii) SKM-1 Naïve and AZA resistant cell lines with and without chloroquine; (B) LD_{50} of MOLM-13 and SKM-1 Naïve with and without chloroquine; (C) ^{14}C-AZA IUR in (i) MOLM-13 (ii) SKM-1 Naïve and AZA resistant cell lines with

and without amantadine; (D) LD_{50} of MOLM-13 and SKM-1 Naïve with and without amantadine. Data represent the mean and all error bars indicate SEM of at least 3 independent experiments. AZA, Azacitidine; M, MOLM-13; S, SKM-1. Unpaired Student's t-test (Welch's correction was applied for data groups with unequal SD) was used to detect statistically significant differences between cohorts. Asterisks display *p*-values * $p < 0.05$, ** $p < 0.01$.

Figure S6.

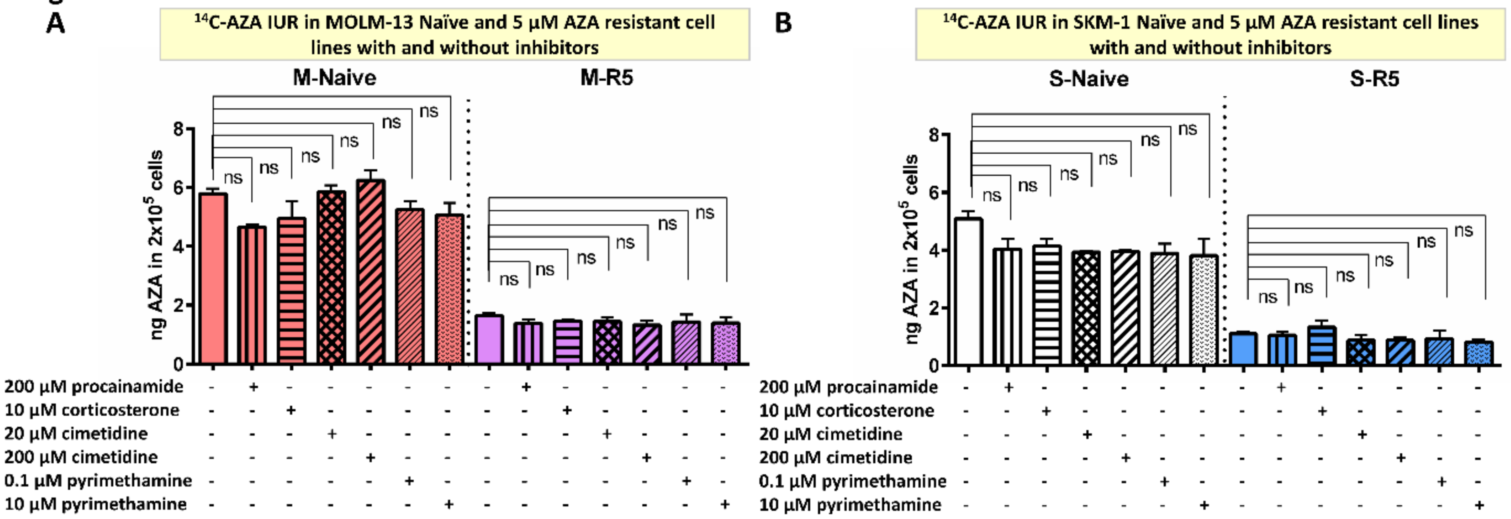

Figure S6. Procainamide, corticosterone, cimetidine and pyrimethamine did not influence the AZA IUR in either Naïve or R5 AZA resistant MOLM-13 (A) and SKM-1 (B) cell lines. Data represent the mean and all error bars indicate SEM of at least 3 independent experiments. AZA, Azacitidine; M, MOLM-13; S, SKM-1.

Unpaired Student's t-test (Welch's correction was applied for data groups with unequal SD) was used to detect statistically significant differences between cohorts.
